# Primary school teachers are immune: a journey in the sea of psychological well-being, buoyancy, and engagement

**DOI:** 10.1186/s40359-024-01592-1

**Published:** 2024-02-21

**Authors:** Zhihan Chen

**Affiliations:** Center for Studies of Education and Psychology of Ethnic Minorities in Southwest China of Southwest University, Chongqing, 400715 China

**Keywords:** Teacher immunity, Psychological well-being, Buoyancy, Engagement, EFL teachers, Primary school

## Abstract

The concept of language teacher immunity is a relatively new notion that has been introduced in the field of language teacher psychology. It is imperative that teachers have their inherent immunity strengthened since they have never been intrinsically protected against fluctuations that are unanticipated and beyond their control. In order to achieve this goal, the present research used a quantitative approach in order to investigate the possible effects of teacher immunity on their psychological well-being, buoyancy, and engagement. This study was conducted by sending out three questionnaires to a total of 384 primary language teachers. On the basis of the findings, it was concluded that teacher immunity has the potential to serve as a significant predictor of English as a foreign language (EFL) teachers’ psychological well-being, buoyancy, and engagement. The research’s conclusions may have substantial implications for education in terms of advancing psychological well-being, buoyancy, and engagement.

## Introduction

Immunity is characterized as a defensive mechanism that stimulates naturally generated defenses and turns down infections via biochemical processes. It functions as a defense mechanism that resists against harmful, undesired, or negative effects of the external environment [1]. In a comparable manner teacher immunity refers to a protective and responsive system, which operates against numerous conflicts and problems in the teaching profession [1, 2]. As [3] defined, teacher immunity is an aggregation of enthusiasm for instruction, emotional health, and receptivity to change on the one extreme and educational demands, exhaustion, and dropout on the other end of the continuum.

Teacher immunity (TI) is centered on self-organization theory that is borrowed from complexity theory [4]. Self-organization describes a mechanism by which the entire operation of a dynamic system changes by means of the collaboration of various components of the structure [5, 6] involving four growth-focused phases: activating, integration, adjustment, and equilibrium [7]. Similar to its roots in biological sciences, teacher immunity is of two sorts: productive immunity and maladaptive immunity [1–3]. As a protective clothing, the first type defends instructors against anxiety, disappointment, exhaustion, and the like. Conversely, the latter adversely influences the instructional mechanisms to make them calcified. Different causes may promote maladaptive immunity, such as avoidance-oriented responses or reluctance to adaptation or novelty [2, 3].

The psychological well-being is a crucial determinant of instructors’ effectiveness that contributes to their students’ achievements. This is because pupils are primarily influenced by the quality of their instructors. Over the past few years, there has been an increased scholarly emphasis on the well-being of educators as a potential mitigating factor against work-related stress and frustration [8]. Teacher psychological well-being (TPW) involves factors such as stress management skills, mental wellness, life satisfaction, and a sense of purpose. A more positive emotional state and enhanced academic performance are both correlates of well-being among students and instructors [9].

TPW encompasses a wide range of positive feelings and states of well-being at the workplace, as well as overall satisfaction with life and one’s career [10]. Researchers in this area generally look at academic and personal factors to determine what makes people happy and successful [11]. Furthermore, there are many different ways to think about wellbeing. Some researchers have pointed out that being able to accept oneself, having a sense of purpose, making progress in one’s life, having supportive relationships, feeling empowered, and having an appreciation for nature are all crucial to flourishing [12]. A TPW significantly affects their ability to interact with students, as has been shown [9]. found that instructors who report greater levels of wellbeing are more engaged and empathetic toward their students, while teachers who report higher levels of emotional exhaustion are more critical of their students.

L2 educational achievement is often characterized as a challenging and stressful setting because to the underlying tensions, disputes, expectations, heavy workloads, and linguistic-cultural disparities and inconsistencies. To keep going even when things become tough in class, teachers need to have a positive attitude [13]. Teacher buoyancy (TB) is a dynamic and changeable aspect of second/foreign language instruction that is affected by intrinsic and extrinsic motivations to succeed. Personality and other internal traits, as well as environmental factors, are known to influence TB [14]. In other words, TB is highlighting strengths rather than weaknesses and being the one to take the lead when things become tough [13]. In other words, a teacher’s TB is their ability to deal with stress in a way that doesn’t hinder their training or performance [15]. Language instructors need to attempt to achieve a balance between their pedagogical expertise and their psychological comprehension of the internal motivations of teaching in order to succeed and operate efficiently [16]. This requires teachers to have buoyancy, which is the ability to effectively handle, endure, and overcome the difficulties encountered in an educational environment [17]. Buoyancy, as described by [18], refers to the positive aspect of resilience that is affected by both internal and external factors.

An engagement-filled work life paves the way to a magnificent career path. Affective motivational theory places a premium on initiative and engagement in the job [19]. According to [20], work engagement is a concept that expresses an individual’s desire for participation and satisfaction in their employment. Put simply, when a person dedicates their whole effort to their job, it is called work engagement [21, 22, 23]. provided the first definition of work engagement as the process of physically, mentally, and emotionally immersing oneself in one’s work duties. In the opinion of [23], people display different levels of their physical, cognitive, and emotional personalities in the roles they take on [23]. introduced the notion of self-in-role, suggesting that people exhibit varying levels of self-expression while engaging in various tasks during their workdays.

Self-determination theory (SDT) lends theoretical credence to the idea of work engagement [24]. According to SDT, workers who are enthusiastic about what they do are more likely to show up to work fresh, persistent, and creative [25, 26]. created a model with three aspects to describe teacher engagement: cognitive-physical, emotional, and social. These dimensions include involvement with students and colleagues. Cognitive-physical engagement refers to the commitment of educators, both mentally and physically, to their work in the classroom. Teachers’ delight and amusement that is in line with education is what is known as emotional involvement [27]. The remaining two components of this approach, which center on the social aspect of teachers’ duties, are social interaction with students and social engagement with colleagues.

### Empirical studies

In accordance to the evaluation of the scant literature on teacher immunity, this path is untapped and asks for more research to provide insight on its correlations with other teacher-related characteristics. For instance, [28] developed a model on the factors influencing language teacher immunity and used a path-analysis method. Their findings suggest that sentimentality, extravagance, and pleasantness have an indirect effect on language teachers’ immunity through job instability and reflective teaching. Additionally, they noted that employment instability had a very negative effect on reflective instruction and language teacher immunity. In the similar vein, [29] conducted research on the most common type of applied immunity strategy among EFL teachers. They found that the most common type of immunity among EFL teachers was maladaptive immunity. They also discovered that EFL teachers established their immunity by beginning, interacting, adjusting, and stabilizing.

Using a similar methodology, [30] showed how favorable the relationship was between the immunity, self-awareness, and work ethic of EFL instructors. In addition, this research requires language teachers to use educational programs in order to increase EFL teachers’ immunity growth, awareness, and engagement. In line with [31], employees who have a high degree of WE tend to be more loyal to their company and motivated at work. In the landscape of primary schools, [32] found that higher acceptance of professional values predicted improvements in professional effectiveness, which indirectly improved teacher engagement.

As a notion best seen in positive psychology, teachers’ psychological wellbeing is described as their assessment and satisfaction with their personal fulfillment, wellness, and career [33]. Numerous research has found that an individual’s level of well-being has a considerable impact on both their psychological condition and their educational practices [9, 12, 34, 35]. stressed that psychological well-being, which includes physical and mental health, life happiness, and work satisfaction, may help people achieve a more permanent sense of purpose. According to [36], in order to be healthy, instructors must be able to deal with work-related stress while still keeping a happy and contented state of mind, establishing relationships, and believing in themselves.

Understanding the elements that contribute to teachers’ well-being is becoming increasingly relevant in educational settings due to the effect of TPW on teacher success, student growth, and academic accomplishment. As such, a multitude of different ideas are linked to the mental health of educators. For example, [37] advocated for the creation of resources to help educators deal with challenges on the job and keep accurate evaluations of their own performance. Teachers who experience positive feelings while teaching might benefit from these materials. However, teachers who deal with negative emotions on the job are more prone to emotional fatigue or disengagement in the classroom, which may lead to a vicious cycle of negative emotions. Consequently, studying what factors affect teachers’ happiness is crucial for students and instructors’ careers, and it may also have positive effects on educational systems throughout across the world.

### Standpoints of this research

Given the scarcity of research in this area and the significance of the aforementioned factors in enhancing language teaching, this study sought to evaluate how TI affects TPW, TB, and TE among EFL teachers. A theoretical framework was developed to demonstrate how TI, TPW, TB, and TE interact with one another. This conceptual framework was built on recent studies and concepts in the field, which were then assessed using Confirmatory Factor Analysis (CFA) and Structural Equation Modeling (SEM), and the findings are discussed. The outcomes of the present investigation have the potential to expand relevant knowledge and research in both empirical and theoretical perspectives. The following questions served as the basis for the research that was conducted in this study:

#### RQ1

Can primary language teacher immunity provide a window into their professional well-being?

#### RQ2

Can primary language teacher immunity provide a window into their buoyancy?

#### RQ3

Can primary language teacher immunity provide a window into their engagement?

## Methodology

The demonstration of the methodological steps is provided in the following:

### Setting and participants

This research included a cohort of 384 EFL instructors, including 171 males and 213 females. The instructors provided education to students in China, who had intermediate proficiency in the English language, teaching in primary schools. The range of teachers’ classroom experience spans from two to twenty-three years, while their ages range from 27 to 44. The bulk of these teachers (*n* = 284) were pursuing a degree in English Education, while a lesser proportion were enrolled in English Literature (*n* = 120), English Translation (*n* = 98), and Linguistics (*n* = 55). Out of the participants, 35 had a Ph.D. degree, and the other participants had either a master’s or bachelor’s degree.

### Instruments

The participants’ immunity was evaluated using the Language Teacher Immunity Instrument (LTII), which [2] designed and certified. There are 39 items in the test, broken down into 7 sub-scales with a 6-point response scale for each (1 being strongly disagree; 6 being strongly agree). Teaching effectiveness (7 items), burnout (5 items), resilience (5 items), attitudes toward teaching (5 items), openness to change (6 items), classroom affectivity (6 items), and coping (5 items) are the sub-scales of this instrument. The LTII’s Cronbach Alpha trustworthiness in this investigation was good, with a range of 0.831 to 0.948.

The psychological well-being observed in teachers was assessed in this study employing the Psychological Well-Being at Work (PWBW) [38] as a measurement instrument. The five underlying elements of this questionnaire—interpersonal fit at work, thriving at work, feeling of competence at work, perceived recognition at work, and desire for involvement at work—all exhibit good reliability coefficients (ranging from 0.824 to 0.877). The scale has 25 statements overall, and each statement is given a 6-point value. The study’s outcomes were corroborated by Cronbach’s alpha, which indicated that the reliability of this scale was good (α = 0.848).

The teacher buoyancy scale, known as the TBS, developed by [17] was used in order to assess the buoyancy of teachers. There are five subscales and 22 items on this scale, which are rated on a Likert scale from one to six. The items are as follows: The following are the items that make up this list: coping with difficulties (six items), bouncing back cognitively and emotionally (six items), working hard and appraising difficulties in a positive manner (three items), caring for one’s own well-being (four items), and striving for professional growth (three items). The results of Cronbach’s alpha indicated that the dependability of each individual component of the TBS was good (varying from 0.841 to 0.892).

The level of teachers’ engagement with their job was evaluated using the Engaged Teacher Scale (ETS) developed by [27]. The instrument comprises 16 items, rated on a seven-point Likert scale (1 = Strongly disagree; 7 = Strongly agree). It consists of four subscales that measure the different aspects of teacher engagement at work: cognitive engagement, emotional engagement, social engagement with students, and social engagement with colleagues. The present research found that the Cronbach’s alpha coefficient indicated adequate levels of reliability for all sub-components of ETS, with values ranging from 0.796 to 0.898.

### Data collection and analysis

This study’s researchers created a web-based platform, namely Google Forms, to aid in data collection over the course of five months in 2023. Evaluating LTII, PWBW, TBS, and ETS are the four aims of this study. To start, the data were checked for normal distribution using the Kolmogorov-Smirnov test. Once it was determined that the data was normally distributed, parametric procedures were suggested for data analysis. To achieve this purpose, LISREL 8.80 was used for SEM and CFA. To evaluate a confirmatory hypothesis-testing approach for the proposed structural theory, SEM was run [39]. All of the latent variables were examined using CFA prior to evaluating a structural model [40].

## Results

A statistical study was performed to investigate the connection between TI, PWB, TB, and TE the findings of that investigation are reported here. There were descriptive data on the TI, PWB, TB, and TE of EFL teachers that are shown in Table 1.

**Table 1:** The results of descriptive statistics

	N	Minimum	Maximum	Mean	Std. Deviation
Teaching self-efficacy	384	7	49	31.628	6.398
Burnout	384	5	35	22.885	4.847
Resilience	384	5	35	22.820	4.451
Attitudes toward teaching	384	5	35	23.341	4.800
Openness to change	384	6	42	27.458	5.107
Classroom affectivity	384	6	42	28.620	4.620
Coping	384	5	35	23.615	4.847
Teacher Immunity	384	121	250	180.367	20.843
Interpersonal Fit at Work	384	5	25	16.456	4.584
Thriving at Work	384	5	25	17.388	5.073
Feeling of Competence at Work	384	5	25	16.617	4.989
Perceived Recognition at Work	384	5	25	17.148	4.650
Desire for Involvement at Work	384	9	25	18.294	3.563
Teacher Psychological Well-being	384	36	123	85.904	17.737
Coping with Difficulties	384	14	30	22.820	4.131
Bouncing Back Cognitively and Emotionally	384	6	36	22.461	4.334
Working Hard and Appraising Difficulties Positively	384	3	18	11.315	3.162
Caring for One’s Well-being	384	11	24	16.414	2.720
Striving for Professional Growth	384	6	18	11.865	2.555
Teacher Buoyancy	384	55	123	84.875	12.836
Cognitive Engagement	384	7	20	13.966	3.817
Emotional Engagement	384	7	20	15.276	2.714
Social Engagement with Students	384	4	20	14.695	3.331
Social Engagement with Colleagues	384	5	20	14.805	2.652
Teacher Engagement	384	30	79	58.742	8.232

The highest mean scores were seen for the constructs teaching self-efficacy (M = 31.628, SD = 6.398) on the TI. The results indicated that desire for involvement at work received the highest level of attention (M = 18.294, SD = 3.563) on the PWB, which was the second instrument. Of all the components of TB, coping with difficulties got the highest average score (M = 22.820, SD = 4.131) as well as bouncing back cognitively and emotionally (M = 22.461, SD = 4.334). In addition, while examining TE, it was evident that emotional engagement (M = 15.276, SD = 2.714) emerged as the prominent frontrunner. For the purpose of determining the effective statistical analysis approach, the Kolmogorov-Smirnov test was conducted.

Table 2 indicates that all of the instruments and their subscales had statistically significant values larger than 0.05. Therefore, parametric approaches are applicable due to the normal distribution of the data.

**Table 2:** The results of Kolmogorov-Smirnov test

	Kolmogorov-Smirnov Z	Asymp. Sig. (2-tailed)
Teaching self-efficacy	1.158	0.137
Burnout	1.010	0.259
Resilience	1.149	0.143
Attitudes toward teaching	1.043	0.227
Openness to change	1.120	0.162
Classroom affectivity	1.200	0.112
Coping	1.457	0.059
Teacher Immunity	0.885	0.413
Interpersonal Fit at Work	0.680	0.744
Thriving at Work	0.884	0.415
Feeling of Competence at Work	0.814	0.521
Perceived Recognition at Work	0.911	0.378
Desire for Involvement at Work	1.204	0.110
Teacher Psychological Well-being	0.753	0.622
Coping with Difficulties	1.146	0.145
Bouncing Back Cognitively and Emotionally	1.030	0.240
Working Hard and Appraising Difficulties Positively	1.285	0.074
Caring for One’s Well-being	1.130	0.155
Striving for Professional Growth	1.160	0.136
Teacher Buoyancy	1.142	0.147
Cognitive Engagement	1.114	0.167
Emotional Engagement	1.384	0.053
Social Engagement with Students	1.419	0.051
Social Engagement with Colleagues	0.666	0.766
Teacher Engagement	0.600	0.864

The researchers examined the relationship TI, PWB, TB, and TE using a Pearson product-moment correlation. In accordance with Table 3, there is a significant and positive correlation between TI, PWB, AB, and TE. Additional information is provided on Table 4.

**Table 3:** The correlation coefficients between the TI, PWB, TB, and TE

	Teacher Immunity	Teacher Psychological Well-being	Teacher Buoyancy	Teacher Engagement
Teacher Immunity	1.000			
Teacher Psychological Well-being	0.854**	1.000		
Teacher Buoyancy	0.725**	0.631**	1.000	
Teacher Engagement	**0.573	**0.658	**0.704	1.000

Correlation is significant at the 0.01 level (2 tailed) **

**Table 4:** The correlation coefficients between the subtitles

	Teaching self-efficacy	Burnout	Resilience	Attitudes toward teaching	Openness to change	Classroom affectivity	Coping	Teacher Psychological Well-being	Teacher Buoyancy	Teacher Engagement
Teaching self-efficacy	1.000									
Burnout	0.534**	1.000								
Resilience	0.502**	0.633**	1.000							
Attitudes toward teaching	0.631**	0.542**	0.598**	1.000						
Openness to change	0.642**	0.668**	0.638**	0.478**	1.000					
Classroom affectivity	0.581**	0.579**	0.670**	0.455**	0.524**	1.000				
Coping	0.564**	0.613**	0.589**	0.635**	0.548**	0.489 **	1.000			
Teacher Psychological Well-being	0.894**	0.789**	0.836**	0.896**	0.804**	0.874 **	0.821**	1.000		
Teacher Buoyancy	0.742**	0.644**	0.778**	0.693**	0.669**	0.672 **	0.712**	0.631**	1.000	
Teacher Engagement	0.558**	0.448**	0.589**	0.641**	0.612**	0.523 **	0.494**	0.658**	0.704**	0.477**

Table 4 demonstrates significant negative correlations between several components of TI, as well as TPW, TB, and TE. Specifically, there were statistically significant positive relationships observed between TPW and Teaching self-efficacy (*r* = 0.894**), Burnout (*r* = 0.789**), Resilience (*r* = 0.836**), Attitudes toward teaching (*r* = 0.896**), Openness to change (*r* = 0.804**), Classroom affectivity (*r* = 0.874**), and Coping (*r* = 0.821**). There was a positive correlation between Teaching self-efficacy (*r* = 0.742**), Burnout (*r* = 0.644**), Resilience (*r* = 0.778**), Attitudes toward teaching (*r* = 0.693**), Openness to change (*r* = 0.669**), Classroom affectivity (*r* = 0.672**), and Coping (*r* = 0.712**). Furthermore, there were robust adverse associations among the subscales of TI and TE: Teaching self-efficacy (*r* = 0.558**), Burnout (*r* = 0.448**), Resilience (*r* = 0.589**), Attitudes toward teaching (*r* = 0.641**), Openness to change (*r* = 0.612**), Classroom affectivity (*r* = 0.523**), and Coping (*r* = 0.494**).

**Fig. 1:**
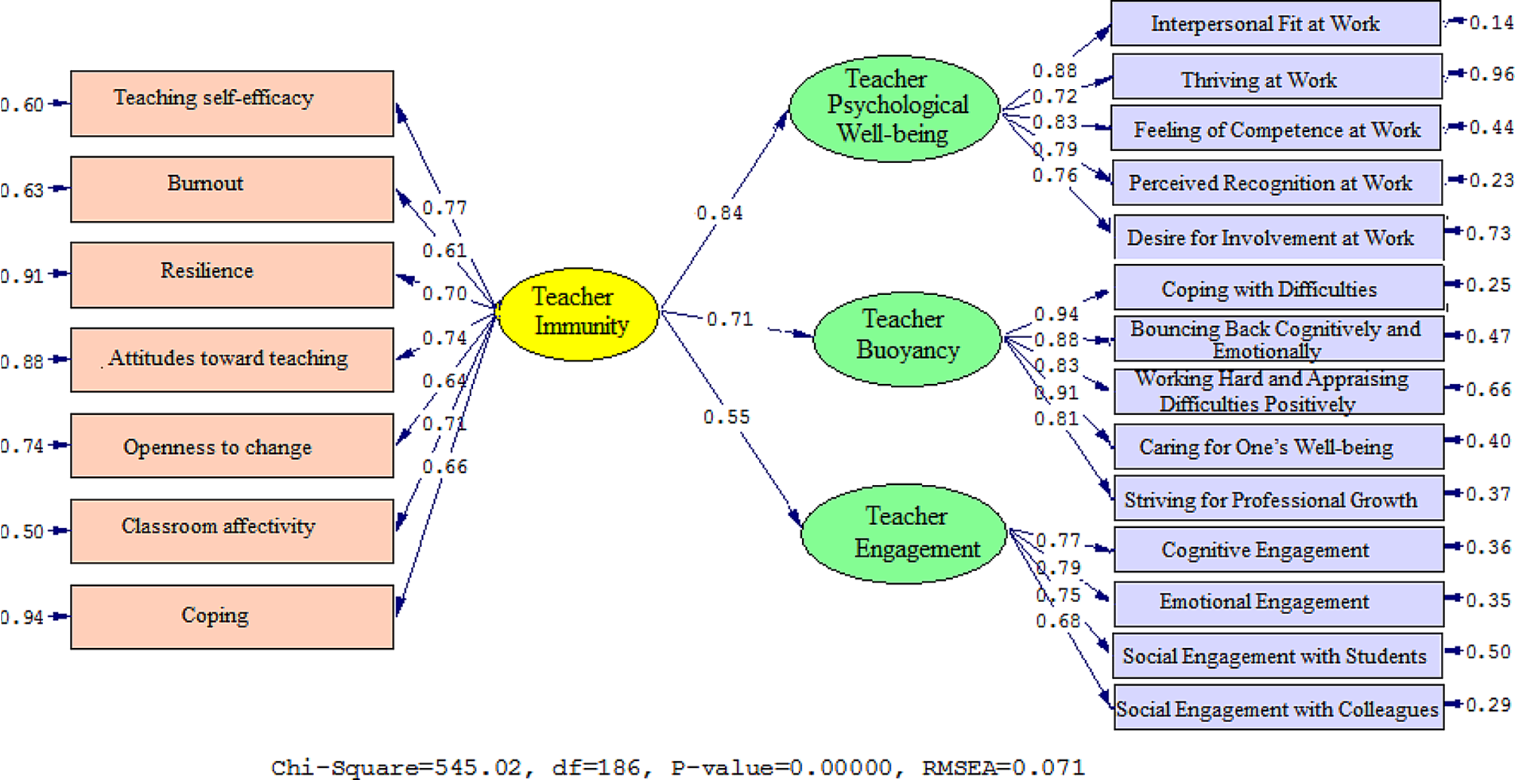
Schematic representation of path coefficient values (Model 1)

**Fig. 2:**
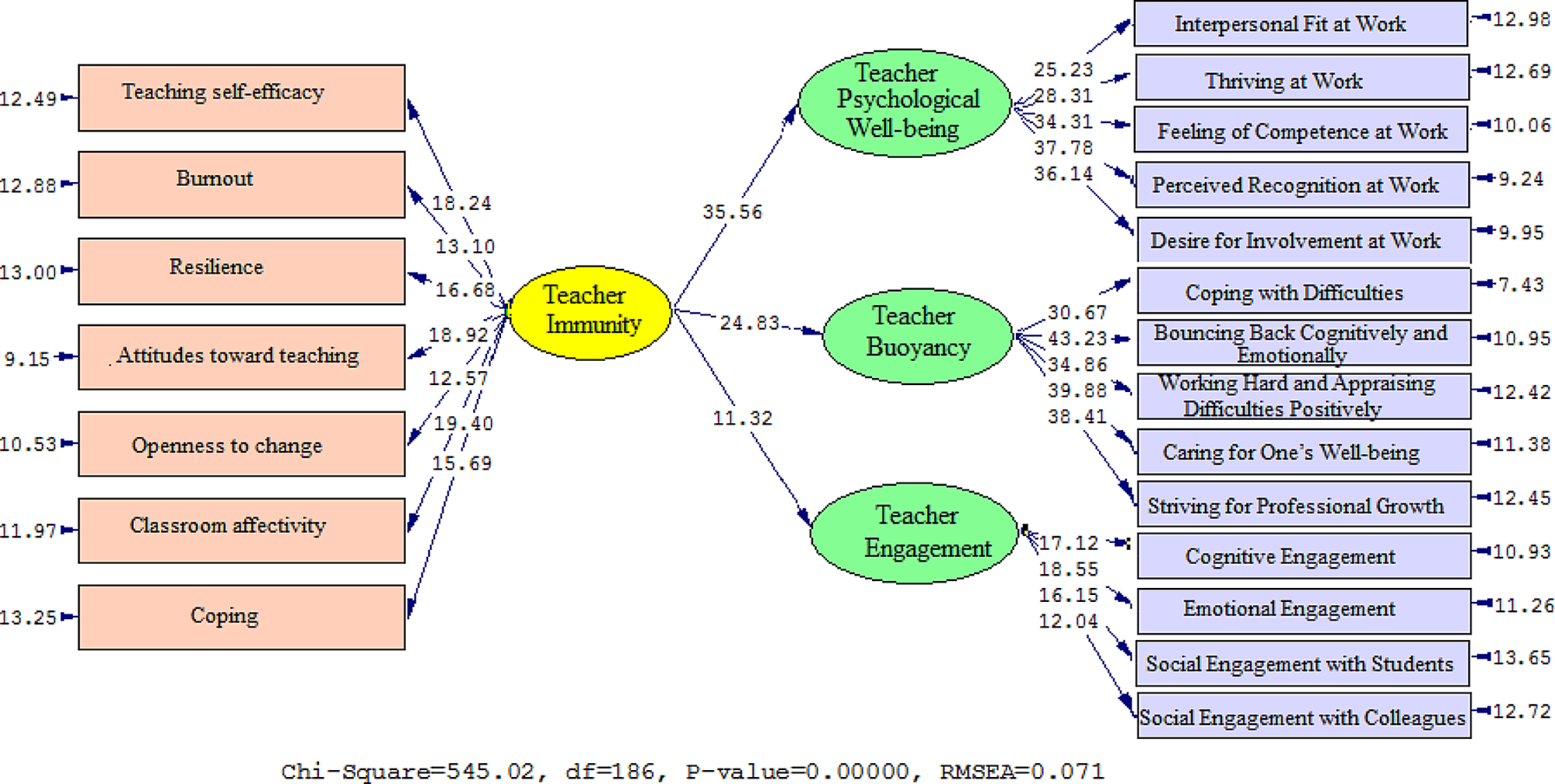
T-values for path coefficient significance (Model 1)

Figures 1 and 2 depict the connection between the variables visually. Table 5 displays standardized estimates and t-values to examine the influence of TI, TPW, TB, and TE. TI influences TPW (β = 0.84, t = 35.56), TB (β = 0.71, t = 24.83), and TE (β = 0.83, t = 25.48).

**Table 5:** Overview of the results in Model 1

Paths			Path Coefficient	T Statistics	Test results
Teacher Immunity	→	Teacher Psychological Well-being	0. 84	35.56	Supported
Teacher Immunity	→	Teacher Buoyancy	0.71	24.83	Supported
Teacher Immunity	→	Teacher Engagement	0.55	11.32	Supported

The model fit was evaluated using the chi-square magnitude, root mean squared error of approximation (RMSEA), goodness of fit (GFI), comparative fit index (CFI), and normed fit index (NFI). According to Jöreskog (1990), the chi-square is considered non-significant, and the chi-square/df ratio should be less than 3. Furthermore, the root mean square error of approximation (RMSEA) must be less than 0.1 (Jöreskog, 1990). A good match is indicated by an NFI cut value larger than 0.90, GFI cut value greater than 0.90, and CFI cut value greater than 0.90 (Jöreskog, 1990).

The findings shown in Table 6 indicate that all of the fit levels for Model 1 were good. The following statistical measures were obtained: the chi-square/df ratio (2.930), RMSEA (0.071), GFI (0.941), NFI (0.956), and CFI (0.933).

**Table 6:** Model fit indices (Model 1)

Fitting indexes	χ^2^	df	χ^2^/df	RMSEA	GFI	NFI	CFI
Cut value			< 3	0.1>	> 0.9	> 0.9	> 0.9
Model 1	545.02	186	2.930	0.071	0.941	0.956	0.933

**Fig. 3:**
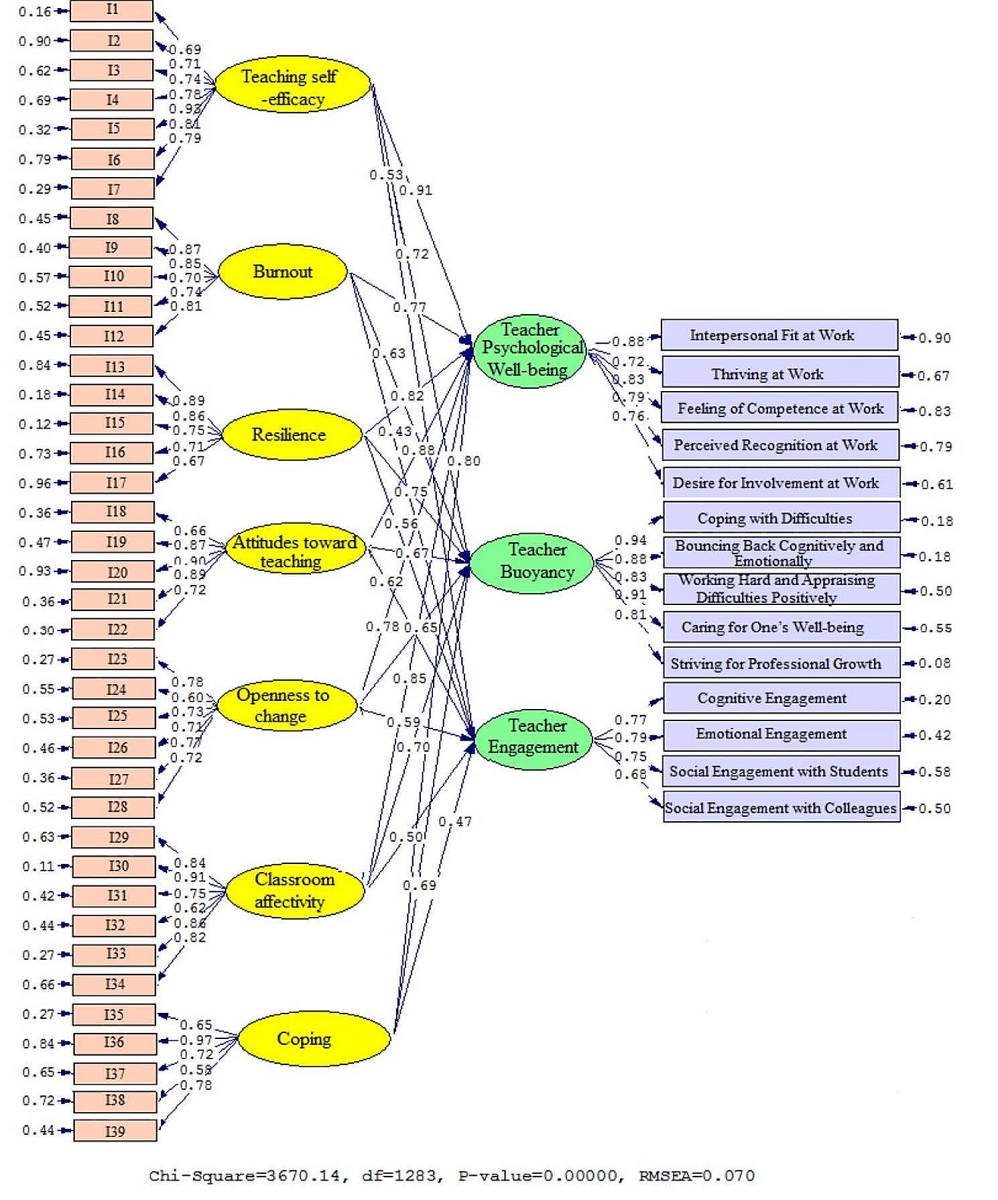
Schematic representation of path coefficient values (Model 2)

**Fig. 4:**
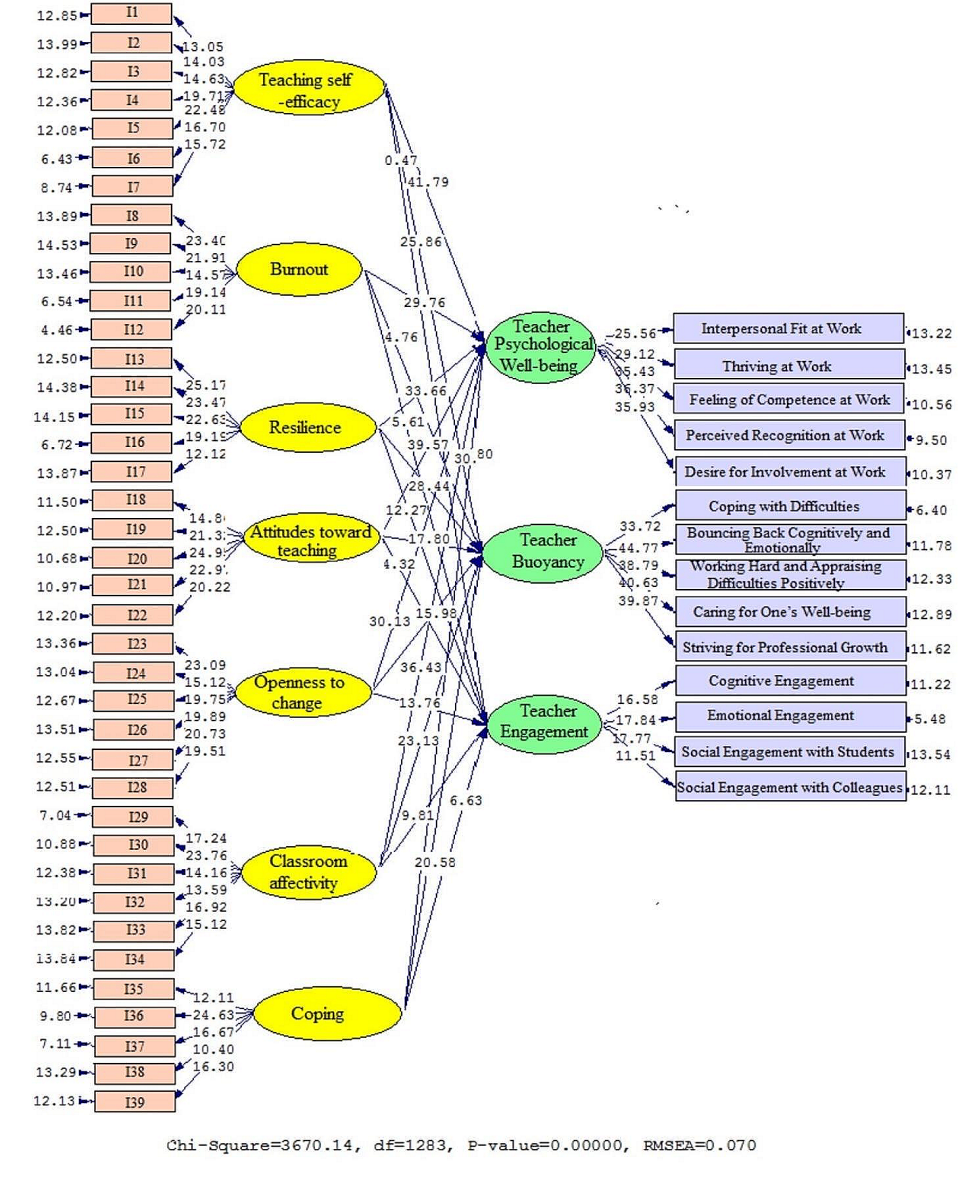
T-values for path coefficient significance (Model 2)

The actual values of the coefficients generated by Model 2 are presented in Figs. 3 and 4 as well as Table 7 to clearly show the connections that were discovered between the subfactors. There was shown to be a link between teaching self-efficacy (β = 0.91, t = 41.79), burnout (β = 0.77, t = 29.79), resilience (β = 0.82, t = 33.66), attitudes toward teaching (β = 0.88, t = 39.57), openness to change (β = 0.78, t = 39.57), classroom affectivity (β = 0.85, t = 36.43), coping (β = 0.80, t = 30.91), and TPW. This is also true when looking at the correlations between teaching self-efficacy (β = 0.72, t = 25.86), burnout (β = 0.63, t = 14.76), resilience (β = 0.75, t = 28.44), attitudes toward teaching (β = 0.67, t = 17.80), openness to change (β = 0.65, t = 15.98), classroom affectivity (β = 0.70, t = 23.13), coping (β = 0.69, t = 20.58), and TB. Furthermore, it was discovered that teaching self-efficacy (β = 0.53, t = 10.47), burnout (β = 0.43, t = 5.60), resilience (β = 0.56, t = 12.07), attitudes toward teaching (β = 0.62, t = 14.32), openness to change (β = 0.59, t = 13.76), classroom affectivity (β = 0.50, t = 9.81), coping (β = 0.47, t = 6.63), and TE were also connected.

**Table 7:** Overview of the results in Model 2

Paths			Path Coefficient	T Statistics	Test results
Teaching self-efficacy	→	Teacher Psychological Well-being	0. 91	41.79	Supported
Burnout	→	Teacher Psychological Well-being	0.77	29.76	Supported
Resilience	→	Teacher Psychological Well-being	0.82	33.66	Supported
Attitudes toward teaching	→	Teacher Psychological Well-being	0.88	39.57	Supported
Openness to change	→	Teacher Psychological Well-being	0.78	30.13	Supported
Classroom affectivity	→	Teacher Psychological Well-being	0.85	36.43	Supported
Coping	→	Teacher Psychological Well-being	0.80	30.91	Supported
Teaching self-efficacy	→	Teacher Buoyancy	0.72	25.86	Supported
Burnout	→	Teacher Buoyancy	0.63	14.76	Supported
Resilience	→	Teacher Buoyancy	0.75	28.44	Supported
Attitudes toward teaching	→	Teacher Buoyancy	0.67	17.80	Supported
Openness to change	→	Teacher Buoyancy	0.65	15.98	Supported
Classroom affectivity	→	Teacher Buoyancy	0.70	23.13	Supported
Coping	→	Teacher Buoyancy	0.69	20.58	Supported
Teaching self-efficacy	→	Teacher Engagement	0.53	10.47	Supported
Burnout	→	Teacher Engagement	0.43	5.60	Supported
Resilience	→	Teacher Engagement	0.56	12.07	Supported
Attitudes toward teaching	→	Teacher Engagement	0.62	14.32	Supported
Openness to change	→	Teacher Engagement	0.59	13.76	Supported
Classroom affectivity	→	Teacher Engagement	0.50	9.81	Supported
Coping	→	Teacher Engagement	0.47	6.63	Supported

The fit indices of the second model are likewise included in Table 8. The RMSEA (0.070) and chi-square (2.861) ratios suggest a successful alignment. Furthermore, the CFI (0.946), NFI (0.935), and GFI (0.955) all fell within acceptable levels.

**Table 8:** Model fit indices (Model 2)

Fitting indexes	χ^2^	df	χ^2^/df	RMSEA	GFI	NFI	CFI
Cut value			< 3	0.1>	> 0.9	> 0.9	> 0.9
Model 2	3670.14	1283	2.861	0.070	0.955	0.935	0.946

## Discussion

Identifying the existence of association between TI, TPW, TB, and TE was the purpose of this research. The findings of this study demonstrated a significant and positive correlation between TI, TPW, TB, and TE among primary language teachers. The result related to the first study question (RQ1: Can primary language teacher immunity provide a window into their professional well-being?) suggest that participants who healthfully and productively immunized would have been better equipped to deal with difficult situations and disagreements at work. The outcomes obtained were in tune with the findings of [7], who highlighted the significance of fostering reflection as a means of increasing TI. To be more specific, the data revealed that the degree of TI-directed perseverance in instruction, passion and purpose in instruction, intrapersonal consciousness, and interpersonal attention. Based on the principles of self-organization theory, productive immunity serves as a defense against a variety of challenges that arise in the workplace [1, 2, 27]. discovered a strong link between language instructors’ immunity and thinking in this respect. It can be argued that higher order cognitive processes promote self-awareness and that self-organization leads to effective immune responses. Self-organization fosters emotional equilibrium, which improves effective immunity and, as a consequence, increases instructors’ commitment to tenacity, excitement, and purpose in the classroom, as well as intrapersonal and interpersonal awareness.

Previous research has found substantial connections between self-efficacy, resilience, burnout, and professional performance (the LTII subsections). The study findings may be attributed to the fact that immunizing teachers allows EFL teachers to get significant insights into all elements of their teaching environment, enabling them to have a strong understanding of the factors that shape their effectiveness [3]. Nevertheless, due to the lack of prior studies directly investigating the relationship between TI and TPI, it is not possible to compare this discovery with others. Thereby, it has the potential to stimulate further investigation in the field of TEFL.

Results from the second study inquiry (RQ2: Can primary language teacher immunity provide a window into their buoyancy?) indicated that the state of TI affects their buoyancy. Furthermore, the data screening in Model 2 displayed that TB, teaching self-efficacy, resilience attitudes toward teaching, openness to change, classroom affectivity, and coping (TI sub-components) are all tightly linked. In a simpler word, buoyant teachers can better cope with difficult situations. Similar to its biological counterpart, immunity shields educators from the ups and downs encountered while pursuing their education [41]. Positive psychology principles may lend credence to this result. As with other areas of positive psychology, language education makes use of self-aid notions to bolster education [42]. Thus, in both intrapersonal and interpersonal mindfulness, instructors who are buoyant are more likely to succeed. In the classroom, they are also less certain and more enthusiastic.

More research is required since the current body of knowledge on TB and TI does not give a clear picture, particularly with regards to EFL in elementary schools. The positive effects of academic buoyancy on the students’ health and performance in the classroom were reflected in the buoyancy domain. The study revealed that the resilience and determination of EFL instructors in the classroom, as well as their feeling of purpose and importance, are enhanced via positive interactions and support from their colleagues. Although there is no conclusive evidence linking TI with TB, the study conducted by [43] suggests that a high level of self-efficacy is associated with greater persistence in the classroom, indirectly supporting this outcome.

Furthermore, it was shown that TI also had a substantial impact on primary language teacher engagement (RQ3: Can primary language teacher immunity provide a window into their engagement?). Immunized instructors are more prone to have a feeling of purpose and meaning in their work. Immunized teachers are more prone to experiencing fulfillment in their career, thereby enhancing their health and happiness. Factors such as self-efficacy in teaching, weariness, persistence, attitudes of teaching, readiness to adaptability, responsiveness in the classroom, and tolerance may all contribute to the occurrence of professional engagement. It seems that teachers who are productive immune, create interesting and effective lesson plans, and have good relationships with students and colleagues are more likely to feel competent and self-assured in their profession [44]. study on primary school teachers in the Netherlands discovered a positive correlation between work engagement and resilience, which is supported by these findings [45]. looked into the causes and effects of meaningful labor in education. Their study’s findings demonstrated a favorable correlation between instructors’ perceptions of fulfilling work and job engagement.

This feeling of ability and trustworthiness might enhance job satisfaction and fulfillment, hence promoting overall psychological well-being [38, 46]. Moreover, when educators see a feeling of autonomy in their profession and possess the competence and resources required to tackle any obstacles that may occur, they are less prone to experiencing burnout and emotional strain [12]. This is due to their superior ability to handle pressures and negotiate challenging circumstances, eventually resulting in enhanced psychological well-being. Teachers who have adaptive immunity are also prone to possess an inclination for enhancement, which exhibits a favorable correlation with their psychological and mental well-being and aids them in coping with stressors [9]. Furthermore, the research findings suggest that teachers who possess immunity exhibit a resolute commitment to achieving their academic goals and attaining success. These results hold considerable promise for the development of programs and initiatives aimed at enhancing teacher well-being in the context of EFL instruction.

## Concluding remarks

The relevance of TI, TPW, TB, and TE is highlighted in the study results, which may aid teacher educators in improving their pre-service and in-service programs. Courses for teacher preparation should also take into account more practical methods to enhance pre-service teachers’ TI, TPW, TB, and TE. As educators’ emotional health has a crucial impact on how they react to reform efforts, it is hoped that the results of this study will motivate language teachers to use techniques for monitoring and managing their own emotions in relation to teaching English in China and other countries. Policymakers are also urged to consider these results in order to have a comprehensive understanding of the factors that contribute to the effectiveness of some programs and teachers and the ineffectiveness of others. Given how new this idea is, politicians, educators, and instructors alike must comprehend the importance of language instructor immunity, particularly among primary teachers.

Schools are recommended to give instructors all the assistance they need to fulfill their responsibilities in order to enhance TI, TPW, TB, and TE. It might take the shape of material assistance, such financing for teachers’ further education or training, or it can take the form of providing and upgrading the facilities used for instruction. Schools should encourage and support teachers to reflect more on their work in order to improve their immunity and self-assessment. Some ways to do this include yearly retreats and recalls and teacher-capability training. To keep raising the standard of language instruction in schools, administrators should be urged to pay more attention to the needs of their instructors and to their requirements. Schools can develop guided mentorship groups for teachers to use as a venue for discussion. It is anticipated that when teachers’ awareness rises, TI, TPW, TB, and TE will rise as well. It is important to support psychological working resources that strengthen reflective teaching methods and advance both teaching and learning. Characteristics like thankfulness, inventiveness, a passion for learning, courage, and others would be among them. With institutional support, courteous leadership built on mutual trust, and adaptation to various working environments, it is possible to foster TI.

Future research may look at some of the limitations of the present study. First, further study is required to improve the generalizability of the findings obtained in various higher education settings throughout the nation, since the individuals were chosen using a convenience sample strategy. As was done in this quantitative investigation, future research may use mixed-methods designs to investigate the relationship between TI, TPW, TB, and TE in order to provide a more comprehensive picture of the issue. Furthermore, because of the cross-sectional nature of the present study, longer-term studies are needed to look at how TI, TPW, TB, and TE. Besides, this study did not look at other explanatory variables such as the demographics of the instructors. Thus, it is recommended that instructors’ demographic data be used in such research in the future. This study was also restricted in Chinese primary schools were English was practiced as a foreign language. Future research can consider the contributions of TI to TPW, TB, and TE in other educational settings. Finally, further research is required to ascertain the extent to which productive immunity, physiological health, buoyant preferences, and engagement may predict the success of their learners.

## Data Availability

The dataset of the present study is available upon request from the corresponding author.

## Abbreviations

EFL: English as a foreign language
TI: Teacher immunity
TPW: Teacher psychological well-being
TB: Teacher buoyancy
SDT: Self-determination theory
CFA: Confirmatory Factor Analysis
SEM: Structural Equation Modeling
LTII: the Language Teacher Immunity Instrument
PWBW: the Psychological Well-Being at Work
TBS: the Teacher Buoyancy Scale
ETS: the Engaged Teacher Scale
RMSEA: the Root Mean Squared Error of Approximation
GFI: the Goodness of Fit
CFI: the Comparative Fit Index
NFI: the Normed Fit Index
